# Efficacy of ethanol-based hand foams using clinically relevant amounts: a cross-over controlled study among healthy volunteers

**DOI:** 10.1186/1471-2334-10-78

**Published:** 2010-03-26

**Authors:** Günter Kampf, Sigunde Marschall, Sven Eggerstedt, Christiane Ostermeyer

**Affiliations:** 1 BODE Chemie GmbH, Scientific Affairs, Melanchthonstr. 27, 22525 Hamburg, Germany; 2 Institut für Hygiene und Umweltmedizin, Ernst-Moritz-Arndt Universität Greifswald, Walther-Rathenau-Str. 49a, 17489 Greifswald, Germany; 3 BODE Chemie GmbH, Development, Melanchthonstr. 27, 22525 Hamburg, Germany; 4 BODE Chemie GmbH, Microbiology, Melanchthonstr. 27, 22525 Hamburg, Germany

## Abstract

**Background:**

Foams containing 62% ethanol are used for hand decontamination in many countries. A long drying time may reduce the compliance of healthcare workers in applying the recommended amount of foam. Therefore, we have investigated the correlation between the applied amount and drying time, and the bactericidal efficacy of ethanol foams.

**Methods:**

In a first part of tests, four foams (Alcare plus, Avagard Foam, Bode test foam, Purell Instant Hand Sanitizer) containing 62% ethanol, which is commonly used in U.S. hospitals, were applied to 14 volunteers in a total of seven variations, to measure drying times. In a second part of tests, the efficacy of the established amount of foam for a 30 s application time of two foams (Alcare plus, Purell Instant Hand Sanitizer) and water was compared to the EN 1500 standard of 2 × 3 mL applications of 2-propanol 60% (v/v), on hands artificially contaminated with *Escherichia coli*. Each application used a cross-over design against the reference alcohol with 15 volunteers.

**Results:**

The mean weight of the applied foam varied between 1.78 and 3.09 g, and the mean duration to dryness was between 37 s and 103 s. The correlation between the amount of foam applied and time until hands felt dry was highly significant (p < 0.001; Pearson's correlation coefficient: 0.724; 95% confidence interval: 0.52-0.93). By linear correlation, 1.6 g gave an intercept of a 30 s application time. Application of 1.6 g of Purell Instant Hand Sanitizer (mean log_10_-reduction: 3.05 ± 0.45) and Alcare plus (3.58 ± 0.71) was significantly less effective than the reference disinfection (4.83 ± 0.89 and 4.60 ± 0.59, respectively; p < 0.001). Application of 1.6 g of water gave a mean log_10_-reduction of 2.39 ± 0.57.

**Conclusions:**

When using 62% ethanol foams, the time required for dryness often exceeds the recommended 30 s. Therefore, only a small volume is likely to be applied in clinical practice. Small amounts, however, failed to meet the efficacy requirements of EN 1500 and were only somewhat more effective than water.

## Background

Alcohol-based hand rubs are recommended for use by healthcare workers for routine decontamination. Based on a tentative final monograph for healthcare antiseptic products [1], most preparations in the U.S. contain between 60% and 70% ethanol, which shows rather poor efficacy on artificially contaminated hands [2,3]. In addition to gel and liquid rubs, ethanol-based foams that also contain 60% to 70% ethanol are available for hand disinfection. Some users report that, compared to liquids or gels, more time is required after foam application until the hands feel dry. Nonetheless, 62% ethanol foams are quite popular among healthcare workers in some countries. Therefore, we investigated 62% ethanol-based foams for correlation between the amount of foam applied, and the time required for drying. We also determined the efficacy of two foams that are often used by healthcare workers in U.S. hospitals, using a standard amount that dried in 30 s which was established from the first series of experiments.

## Methods

The first part of the study included four different 62% ethanol foams. Three of them represent the majority of hand-disinfecting foams commonly used by healthcare workers in the U.S., and one is a test product. The manufacturers were in alphabetical order Bode Chemie GmbH (Hamburg, Germany), Gojo Industries Inc. (Akron, OH, USA), Steris Corporation (St. Louis, MO, USA), and 3M (St. Paul, MN, USA).

A panel of 14 subjects applied the foams using various methods (Table 1). Alcare plus recommends a golf-ball sized amount of foam. The others gave general recommendations such as "a sufficient amount" or "enough product". Whenever such a general description was provided, the foams were tested in the same way as Alcare plus, to simulate as closely as possible what manufacturers recommend. Seven application variations were tested. For Alcare plus, a golf ball-sized amount of foam was applied using an actual golf ball for reference, and rubbed into both hands (n = 14). All four foams (Alcare plus, Avagard Foam, Bode test foam, Purell Instant Hand Sanitizer) were tested by applying a golf ball-sized amount of foam, but without the reference golf ball, and rubbing into both hands (n = 56). For Purell Instant Hand Sanitizer, two other variations were investigated, with application by either three or four applicator pumps, and rubbing into both hands (n = 28). All foams were applied without a specific instruction on the rub-in technique. Subjects had to ensure that both hands are completely covered which has been shown to yield a better coverage of hands compared to the six steps described in EN 1500 [4].

**Table 1:** Label recommendations for application of four different 62% ethanol foams, their mode of application in the study with 14 subjects per type of application, and mean weight and mean drying time.

Foam	Label recommendation	Mode of application	Weight of the applied amount of foam (mean ± stdev)	Time to dry (mean ± stdev)
Alcare plus	Dispense a palmful (golf ball) in one hand. Spread over both hands up to one-half inch above the wrists. Rub vigorously.	Apply a golf ball-sized amount of foam using a golf ball as reference, rub into both hands	3.09 ± 0.63 g	103 ± 34 s
		
		Apply a golf ball-sized amount of foam with no reference golf ball, rub into both hands	2.56 ± 0.81 g	78 ± 30 s

Avagard Foam	Apply sufficient amount to thoroughly wet all surfaces of hands and fingers. Rub onto hands until dry.	Apply a golf ball-sized amount of foam with no reference golf ball, rub into both hands	1.99 ± 0.93 g	60 ± 30 s

Bode test foam	Not available.	Apply a golf ball-sized amount of foam with no reference golf ball, rub into both hands	2.16 ± 0.52 g	80 ± 34 s

Purell Instant Hand Sanitizer	Place enough product in your palm to thoroughly cover your hands. Rub hands together briskly until dry.	Apply a golf ball-sized amount of foam with no reference golf ball, rub into both hands	1.98 ± 0.42 g	57 ± 18 s
		
		Pump applicator three times and rub into both hands	1.78 ± 0.04 g	37 ± 10 s
		
		Pump applicator four times and rub into both hands	2.38 ± 0.05 g	63 ± 19 s

Each foam was weighed on a watch glass before immediate transfer to the subjects' hands. The empty watch glass was weighed again, and the difference recorded as the applied amount of foam applied. The subject spread and rubbed the foam over both hands, noting the time required until the hands felt dry again. For each foam and application variation, the mean application duration and mean foam weight were calculated. A linear correlation between the duration and the weight of foam was evaluated for all variations, to identify the amount of foam likely to keep hands the wet for 30 s.

The second part of the study determined the efficacy of two foams (Alcare plus, Purell Instant Hand Sanitizer) according to EN 1500 [5] which were randomly selected out of the three commercially available hand foams. Briefly, the bactericidal efficacy of each foam was compared to 2-propanol 60% (v/v) in three separate cross-over experiments on the artificially contaminated hands of 15 volunteers. In each experiment subjects were randomly assigned to receive either foam or reference as the first application, with eight volunteers receiving foam first, and seven receiving the reference alcohol first. As per cross-over design, in the second application after approximately 3 h, the subjects received the other product.

For artificial contamination, hands were washed for one min with soft soap, dried with paper towels, immersed in the contamination fluid up to the mid-metacarpals for 5 s with fingers spread, and allowed to dry for 3 min [6]. To determine pre-decontamination values, fingertips were rubbed for one min in a petri dish containing liquid broth. Either 1.6 g of foam, 1.6 g of water, or 2 × 3 mL of reference alcohol were applied to the hands. Foams and water were rubbed into the hands for 30 seconds, and reference alcohol for 60 s. The 60 s application time and the 2 × 3 ml volume for reference alcohol do not reflect clinical practice, but are well-accepted standards for determining the minimum efficacy of hand disinfectants in healthcare [3,6-8]. The EN 1500 handrubbing technique was used [5]. Post-decontamination values were determined immediately after the rub-in period using petri dishes containing liquid broth with neutralisers (3% Tween 80, 0.3% lecithin, 0.1% histidine, 0.1% cysteine). For both reference and test products, log counts from the left and right hands of each subject were averaged separately, for both pre-values and post-values. The arithmetic means of all individual log_10 _reduction values were calculated. The Wilcoxon matched-pairs signed rank test (one-sided) was used for pair-wise comparison between mean log_10 _values obtained with foam or water and the reference alcohol (significance level, p = 0.01).

## Results

**Figure 1:**
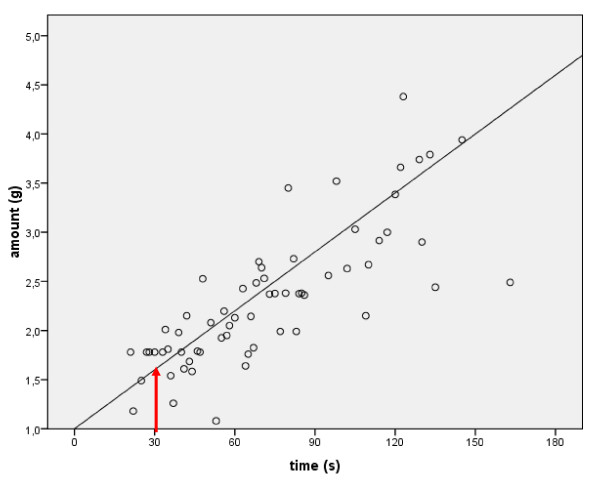
**Correlation between the applied amount of 62% ethanol foam and the time required for hands to feel dry; the red arrow indicates the intercept between a drying time of 30 s and the corresponding weight of foam**.

The drying time of four different foams was evaluated in seven variations with 14 subjects per test run (Table 1). The mean weight of applied foam varied between 1.78 and 3.09 g, and the mean duration to dryness was between 37 s and 103 s. When a golf ball was visible to the user, the mean amount of applied foam was larger (3.09 g versus 2.56 g), and the mean time to dryness was longer (103 s versus 78 s). The correlation between the applied amount of foam and the time until hands felt dry was highly significant (p < 0.001; Pearson's correlation coefficient: 0.724; 95% confidence interval: 0.52-0.93) (Fig. 1). The linear correlation described by the formula y = 0.02*x + 1 showed that an amount of 1.6 g gave an intercept of 30 s application time, which is the time necessary to ensure an adequate quality of hand coverage [4].

The efficacy of two foams (Alcare plus and Purell Instant Hand Sanitizer) was determined according to EN 1500, using 1.61 ± 0.02 g per application (Alcare plus) and 1.60 ± 0.01 g (Purell), and a 30 s application time. Both foams were significantly less effective than the reference procedure of 2 × 3 mL applications of 60% isopropanol for 60 s (Table 2), and thus failed to meet the European efficacy requirements for hygienic hand disinfection. The effect of the foams, expressed as the log_10 _difference from the reference procedure, was only 0.66 log_10 _higher for Alcare plus, and 1.19 log_10 _higher for Purell Instant Hand Sanitizer, than 1.6 g of water applied for 30 s.

**Table 2:** Efficacy expressed as mean log_10_-reduction with stdev of two 62% ethanol foams or water, compared to EN 1500 reference disinfection of 2 × 3 mL 60% isopropanol.

Product (all 1.6 g per application)	Product	EN 1500 reference treatment	p-value
Purell Instant Hand Sanitizer	3.05 ± 0.45	4.83 ± 0.89	≤ 0.001

Alcare plus	3.58 ± 0.71	4.60 ± 0.59	≤ 0.001

Water (negative control)	2.39 ± 0.57	4.54 ± 1.01	≤ 0.001

## Discussion

Ethanol-based foams for hand decontamination have gained attention among healthcare professionals because they stay on the hands, and allow good visual coverage. When ethanol-based foams were applied according to manufacturer's instructions, and a golf ball-sized amount was used, the time required for the hands to feel dry was between 40 s and 90 s, which might be inappropriate for clinical practice, since time constraints are regarded as the major obstacle for high compliance in hand hygiene [9,10]. Consequently, healthcare workers will be tempted to apply a smaller amount, but application of the amount of foam that allowed the hands to feel dry after 30 s was low, at 1.6 g. This amount gave a poor efficacy, similar to the efficiency of gels with the same concentration of ethanol [3], and was only slightly better than rubbing with water alone. The effect of rubbing in 1.6 g water alone reduced test bacteria by 2.4 log_10 _but this result might be because some bacteria were removed by the baseline sampling before water application, and some were removed by the 5 s rinse after application and before sampling. In addition, hands were contaminated up to the metacarpals, and rubbing with 1.6 g of water spread the test bacteria over the hands, reducing the number on the fingertips.

Since the observed efficacy of 1.6 g of ethanol-based foams was only slightly improved over the application of the same amount of water, they cannot be recommended for hospital use. The ability of this product to provide sufficient patient safety is questionable. Healthcare workers are likely to apply an amount of foam that does not keep their hands moist for sufficient clinical efficacy. Compared to published data, even a simple hand wash has a similar or better antimicrobial efficacy as 1.6 g of 62% ethanol foam [8]. As with gels, a higher concentration of ethanol might improve the efficacy of foams [11]. That is why other foams may reveal a better efficacy with a 1.6 g application.

Our data were obtained in a laboratory setting and not under clinical conditions, so the test situation is a limitation of this study. In addition, the level of log_10 _reduction on hands to prevent nosocomial infections is under scientific debate. Nevertheless, a recent controlled prospective cross-over trial in intensive care units showed that introduction of a gel-based 62% ethanol product might improve compliance. The incidence of healthcare-associated infections, however, remained unchanged [12], suggesting that the concentration of ethanol in the gel may have been too low to prevent cross-transmission in clinical practice. A hand rub with a better log_10 _reduction on hands, however, was shown to prevent nosocomial infections [13]. This supports our concerns about the efficacy of foams based on 62% ethanol.

One of the foams was applied as three and four pumps of the applicator (Table 1), for mean amounts of 1.78 g, and 2.38 g, respectively. If healthcare workers pump only once, the dispensed amount could be as small as 0.6 g. Even two pumps would be less than 1.6 g per application. Based on these data, the amount of foam recommended on product labels for the post-contamination treatment of hands should be more precise, and address both the efficacy and a clinically acceptable time for drying after application. Otherwise, the use of the investigated 62% ethanol foams should be critically reviewed in hospitals, as they may jeopardize patient safety. More data with foams and their efficacy should be available in the future, preferably under clinical conditions.

## Conclusions

When using 62% ethanol foams, the time required for dryness often exceeds the recommended 30 s. Therefore, only a small volume is likely to be applied in clinical practice. Small amounts, however, failed to meet the efficacy

## Competing interests

All authors are employed by Bode Chemie GmbH, Hamburg, Germany.

## Authors' contributions

GK, SM and SE made substantial contributions to conception and design, SM and CO made a substantial contributions to acquisition, analysis and interpretation of data. GK was involved in drafting the manuscript, and all authors gave final approval of the version to be published.

## Pre-publication history

The pre-publication history for this paper can be accessed here:

http://www.biomedcentral.com/1471-2334/10/78/prepub

## References

[B1] Anonym Tentative final monograph for health care antiseptic products; proposed rule Fed Reg 1994 59 116 31401 31452

[B2] Kampf G How effective are hand antiseptics for the post-contamination treatment of hands when used as recommended? Am J Infect Control 2008 36 5 356 360 10.1016/j.ajic.2007.07.017 18538702

[B3] Kramer A Rudolph P Kampf G Pittet D Limited efficacy of alcohol-based hand gels Lancet 2002 359 1489 1490 10.1016/S0140-6736(02)08426-X 11988252

[B4] Kampf G Reichel M Feil Y Eggerstedt S Kaulfers P-M Influence of rub-in technique on required application time and hand coverage in hygienic hand disinfection BMC Infect Dis 2008 8 149 10.1186/1471-2334-8-149 18959788 PMC2600642

[B5] EN 1500 Chemical disinfectants and antiseptics. Hygienic hand disinfection. Test method and requirement (phase 2, step 2) 1997 Brussels: CEN - Comité Européen de Normalisation

[B6] Kampf G Ostermeyer C Inter-laboratory reproducibility of the EN 1500 reference hand disinfection J Hosp Infect 2003 53 4 304 306 10.1053/jhin.2002.1357 12660128

[B7] Kampf G Rudolf M Spectrum of antimicrobial activity of Sterillium Gel, a new ethanol-based handgel12th Annual Scientific Meeting: 2002; Salt Lake City, USA 2002

[B8] Kampf G Ostermeyer C Intra-laboratory reproducibility of the hand hygiene reference procedures of EN 1499 (hygienic hand wash) and EN 1500 (hygienic hand disinfection) J Hosp Infect 2002 52 3 219 224 10.1053/jhin.2002.1299 12419275

[B9] Pittet D Improving adherence to hand hygiene practice: a multidisciplinary approach Emerg Infect Dis 2001 7 2 234 240 10.3201/eid0702.010217 11294714 PMC2631736

[B10] Voss A Widmer AF No time for handwashing!? Handwashing versus alcoholic rub: can we afford 100% compliance? Infect Control Hosp Epidemiol 1997 18 3 205 208 10.1086/647590 9090551

[B11] Kampf G Rudolf M Labadie J-C Barrett SP Spectrum of antimicrobial activity and user acceptability of the hand disinfectant agent Sterillium Gel J Hosp Infect 2002 52 2 141 147 10.1053/jhin.2002.1281 12392906

[B12] Rupp ME Fitzgerald T Puumala S Anderson JR Craig R Iwen PC Jourdan D Keuchel J Marion N Peterson D Prospective, controlled, cross-over trial of alcohol-based hand gel in critical care units Infect Control Hosp Epidemiol 2008 29 1 8 15 10.1086/524333 18171181

[B13] Pittet D Hugonnet S Harbarth S Monronga P Sauvan V Touveneau S Perneger TV Effectiveness of a hospital-wide programme to improve compliance with hand hygiene Lancet 2000 356 1307 1312 10.1016/S0140-6736(00)02814-2 11073019

